# Mini-Mental State Examination for telephone use in highly educated and socially active older adults: a descriptive study

**DOI:** 10.1590/1980-5764-DN-2025-0365

**Published:** 2026-01-23

**Authors:** Thaís Bento Lima da Silva, Gabriela dos Santos, Tiago Nascimento Ordonez, Sabrina Aparecida da Silva, Maria Antônia Antunes Fernandes, Wellington Lourenço Oliveira, Diana dos Santos Bacelar, Laydiane Alves Costa, Ana Paula Bagli Moreira, Edna Letícia Queiroz, Gabriela Cristina Siqueira, Rosa Yuka Sato Chubaci, Henrique Salmazo da Silva, Beatriz Aparecida Ozello Gutierrez, Sonia Maria Dozzi Brucki

**Affiliations:** 1 Universidade de São Paulo, Faculdade de Artes, Ciências e Humanidades, Departamento de Gerontologia, São Paulo SP, Brazil.; 2 Universidade Federal do Recôncavo da Bahia, Faculdade de Medicina, Centro de Ciências Médicas, Santo Antônio de Jesus BA, Brazil.; 3 Universidade de Santo Amaro, Faculdade de Medicina, São Paulo SP, Brazil.; 4 Universidade de São Paulo, Faculdade de Medicina, Hospital das Clínicas, Grupo de Neurologia Cognitiva e do Comportamento, São Paulo SP, Brazil.

**Keywords:** Cognition, Aged, Mental Status and Dementia Tests, Dementia, Cognição, Idoso, Testes de Estado Mental e Demência, Demência

## Abstract

With population aging, the growing concern about cognitive decline and dementias highlights the need for early and accessible cognitive assessment. In this context, a telephone-adapted version of the Mini-Mental State Examination, the Brazilian Telephone Version of the Mini-Mental State Examination (Braztel-MMSE), was developed.

**Objective:**

The aim of the study was to compare cognitive performance assessed by the Braztel-MMSE, administered by telephone, with the traditional Mini-Mental State Examination (MMSE), administered in person, in highly educated older adults without diagnosed dementia or depression, and who are socially engaged.

**Methods:**

This is a descriptive cross-sectional study. The initial sample consisted of 578 older adults who completed the Braztel-MMSE during the screening phase of a clinical trial. Of these, a subsample of 190 older adults completed the traditional MMSE after 18 months. Data were analyzed descriptively and inferentially to compare scores obtained from both instruments.

**Results:**

The results showed a significant correlation between scores on the Braztel-MMSE and the traditional MMSE (Spearman's rho=0.354, p<0.001). Bland-Altman analysis revealed satisfactory agreement between the two cognitive assessment methods, with a mean difference of 0.002 between scores.

**Conclusion:**

This study demonstrates that the telephone-administered Braztel-MMSE shows good agreement with the traditional face-to-face MMSE. These findings suggest that the Braztel-MMSE can be a useful and valid tool for cognitive screening in contexts where in-person evaluation is not feasible, including long-term clinical trials.

## RESUMO.

Com o envelhecimento da população, a crescente preocupação com o declínio cognitivo e as demências evidencia a necessidade de uma avaliação cognitiva precoce e acessível. Nesse contexto, surge uma versão do Miniexame do Estado Mental, adaptada para aplicação por telefone (Braztel-MMSE).

**Objetivo:**

Comparar o desempenho cognitivo avaliado pelo Braztel-MMSE, aplicado por telefone, com o Miniexame de Estado Mental (MEEM) tradicional, aplicado presencialmente, em idosos de alta escolaridade, sem diagnóstico de demência e depressão e engajados socialmente.

**Métodos:**

Trata-se de um estudo descritivo de corte transversal. A amostra foi composta inicialmente de 578 idosos que responderam ao Braztel-MMSE na etapa de triagem do ensaio clínico. Desses idosos, uma subamostra de 190 pessoas realizou o MEEM tradicional após 18 meses. Os dados coletados foram analisados de maneira descritiva e inferencial, entre as pontuações obtidas nos dois instrumentos.

**Resultados:**

Os resultados demonstraram correlação significativa entre as pontuações obtidas no Braztel-MMSE e no MEEM tradicional (rho de Spearman=0.354, p<0.001). A análise de Bland-Altman revelou concordância satisfatória entre os dois métodos de avaliação cognitiva, com média de diferença de 0,002 entre as pontuações.

**Conclusão:**

Este estudo evidenciou que o Braztel-MMSE, aplicado por telefone, apresenta boa concordância com o MEEM tradicional, realizado presencialmente. Esses resultados sugerem que o Braztel-MMSE pode ser uma ferramenta útil e válida para a triagem cognitiva em contextos nos quais a avaliação presencial não seja viável, incluindo em ensaios clínicos de longo prazo.

## INTRODUCTION

The number of people with dementia has continued to rise, largely due to the aging of the population. In 2019, an estimated 57 million people were living with dementia, a figure set to increase to around 153 million by 2050^1^. Amid this scenario, discussions and support regarding the risk factors for developing the disease are of the utmost importance, where knowledge and action on these factors can help change this outlook.

According to Caramelli and Resende^2^, dementia can be defined as a syndrome characterized by cognitive decline with behavioral changes that impair individual functioning. Diagnosing dementia is challenging, requiring a thorough anamnesis and assessment of cognitive performance and functioning of the patient. In some cases, complementary exams, such as blood tests and electroencephalograms (EEGs), may be ordered to help confirm diagnosis^2^.

Applying cognitive screening tools is essential for early detection of functional decline, particularly in older adutls^3^. The Mini-Mental State Examination (MMSE; Supplementary Material 1) is one of the most commonly used tests for cognitive screening and is widely applied in clinical and research settings for its simplicity and ability to measure multiple aspects of cognition^4^.

However, applying the exam in person can pose logistical challenges, particularly in regions with poor access to health services or in situations where patient mobility is limited^5^. These challenges create the need for alternatives that allow remote assessment and maintain the quality and accuracy of results^6^.

In this context, the Brazilian version of the MMSE adapted for a telephone application (Braztel-MMSE; Supplementary Material 2) represents a viable solution to overcome these difficulties. This version of the scale retains the same structure as the original scale but provides the necessary flexibility for remote administration, improving access to cognitive screening in situations where in-person evaluation is not practicable^5^.

Studies have demonstrated the validity of this approach, suggesting that the tool is effective for screening cognitive performance, especially in populations with difficulties accessing healthcare or engaged in long-term clinical trials^7,8^.

Despite the advantages of this solution, its efficacy in specific groups, such as socially engaged highly educated older adults, has yet to be explored. Active individuals with a higher educational level generally have greater cognitive reserve, possibly masking early signs of decline^9^.

While both the traditional exam and its adapted version have been validated in different populations, studies focused specifically on socially engaged highly educated older adults are scarce. Comparing the performance of the two assessment approaches (in-person vs. remote) in individuals with these characteristics can provide a deeper understanding of the applicability of the telephone version in more specific contexts. In addition, this analysis can serve to inform discussions on the sensitivity and accuracy of the tool for early detection of signs of cognitive decline.

Therefore, the objective of the present study was to compare the cognitive performance of highly educated older adults without dementia or depression on the telephone and traditional versions of the MMSE. The analysis sought to determine the validity and reliability of the adapted scale and investigate its sensitivity for detecting early cognitive impairments, particularly in individuals with greater cognitive reserve.

The results of the comparison between the two versions provide evidence supporting the applicability of the scale for longitudinal clinical trials and its potential as a cognitive screening tool in the context of population aging.

## METHODS

### Participants

This study focused specifically on socially engaged, highly educated older adults, a subgroup characterized by greater cognitive reserve. Higher education and active social engagement can mask early signs of cognitive decline, making detection more challenging. Assessing the validity of the telephone-based MMSE in this group is therefore important to determine the tool's sensitivity in populations where mild impairments are typically harder to identify.

**Figure 1:**
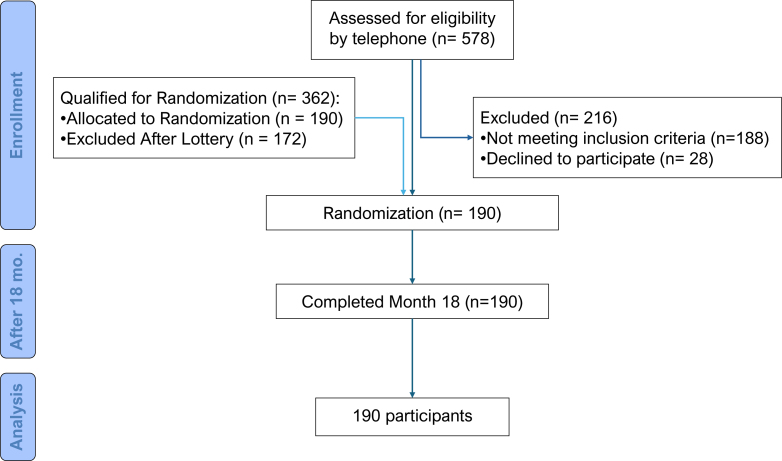
Consolidated Standards of Test Reporting (CONSORT) diagram.

The study sample comprised older adults (age≥60 years) residing in São Paulo city who were users of hospital-run community centers and pensioners’ associations, recruited between February and September 2021. The initial sample consisted of 578 individuals who underwent screening using the BRAZTEL-MMSE. After applying the exclusion criteria, 190 participants were randomly selected and underwent the traditional MMSE, enabling comparison of scores obtained on the respective versions (Figure 1).

These participants were drawn from a larger 24-month randomized controlled trial evaluating a multifactorial cognitive stimulation program in older adults without dementia or depression. From the original pool of 578 recruited individuals, 362 were eligible after initial screening, and a subset of 255 participants was randomly selected for inclusion due to logistical constraints. Stratified randomization (by sex, age, and education) assigned participants to three groups: Training Group, Active Control Group, and Passive Control Group, each initially with 85 participants. Following allocation, 48 individuals declined participation, resulting in the final sub-sample of 190 participants who completed both the telephone BRAZTEL-MMSE and the in-person MMSE, forming the dataset analyzed in this study.

During initial screening, participants scoring>5 points on the Geriatric Depression Scale^10^ or below the cut-off on the BRAZTEL-MMSE were excluded^5^. Further exclusion criteria were individuals with visual, auditory, or motor deficits precluding comprehension of the instructions or execution of the cognitive tasks. In addition, individuals with non-controlled chronic diseases, such as systemic arterial hypertension or diabetes mellitus, or with severe psychiatric disorders, including severe depression, schizophrenia, or bipolar disorder, were also excluded. Information on these aspects was self-reported.

Lastly, participants reporting clinical evidence or neuroimaging findings that indicated vascular disease, as well as individuals with a prior diagnosis of dementia or cognitive disorder, were also excluded.

### Experimental procedures

The BRAZTEL-MMSE was administered first, during the initial screening phase, while the in-person MMSE was conducted 18 months later. This interval reflected the design of the larger clinical study from which the sample was derived.

Sociodemographic information was collected using a structured questionnaire probing sex, marital status, education, and employment/benefits status (pension, retirement, or Continuous Cash Benefit [BPC]). For the cognitive assessment, the versions of the Brazilian traditional MMSE and telephone BRAZTEL-MMSE were applied.

The MMSE provides a measure of general cognitive functioning of an individual, with a focus on domains of memory, orientation for time and space, attention, language, and visuospatial capacity. The scale comprises a series of questions and simple tasks, such as naming an object, repeating phrases, and performing calculations. The maximum score on the MMSE is 30 points, and a score<24 generally indicates some level of cognitive impairment, suggesting the need for more in-depth assessment.

The BRAZTEL-MMSE is a 22-item telephone version of the Brazilian MMSE with a cut-off of 15 points^5^. The assessment protocol was applied by duly trained psychologists and gerontologists.

### Ethics declaration

This study was approved by the Research Ethics Committee of the Clinicas Hospital of the School of Medicine of the University of São Paulo under permit no. 4.357.429 (CAAE:35462620.2.0000.0068) and conducted in accordance with the precepts of the Declaration of Helsinki. All participants were given an explanation of the study goals and protocol prior to signing the Free and Informed Consent Form.

### Statistical analyses

Descriptive statistics were used to characterize the sample based on key demographic variables, including sex, age, education, marital status, and retirement status. Measures of central tendency and dispersion (mean, standard deviation, median, and range) were calculated for continuous variables such as age, education, and cognitive scores. Frequency distributions were analyzed for categorical variables and cognitive test performance.

The statistical analyses in this study focused on describing the sample and comparing cognitive performance across the telephone (BRAZTEL-MMSE) and traditional versions of the MMSE. Initially, descriptive statistics were used to outline the sociodemographic profile of the participants. The convergent validity between the two instruments was assessed using Spearman's rank correlation coefficient, given the ordinal nature of the scores and the lack of assumption of normality. Furthermore, agreement between the two versions was evaluated using Bland-Altman analysis, which allowed for visualization of the distribution of individual differences in scores.

## RESULTS

The final sample comprised 190 older adults aged 60–84 (mean=67.32±4.93) years and a mean education of 17±4.77 years (Table 1). The study participants were predominantly female (76.31%). Most participants declared their marital status as married/civil union (48.95%), followed by single, separated/divorced (14.74%), and widowed (13.16%). Most participants claimed benefits, pension, retirement, or BPC (85.26%).

**Table 1:** Descriptive statistics for the overall sample of 190 participants.

Variables		n=190	%	Mean	SD	Min.	Median	Max.
Sex	Female	145	76.32					
	Male	45	23.68					
Age		190	100.00	67.32	4.93	60.00	67.00	84.00
Education		190	100.00	17.09	4.77	8.00	16.00	45.00
Marital status	Widowed	25	13.16					
	Married/Civil Union	93	48.95					
	Single	44	23.16					
	Separate/divorced	28	14.74					
Pension or retirement	Yes	162	85.26					
	No	28	14.74					

Abbreviations: SD, standard deviation; Min., minimum; Max., maximum.

Participants had mean scores of 20.47±1.39 on the BRAZTEL-MMSE and 28.52±1.56 on the MMSE (Table 2). Total scores on the BRAZTEL-MMSE ranged from 15 (minimum) to 22 (maximum) points, with the highest prevalence rates of 21 points and 22 points. Total scores on the MMSE ranged from 23 (minimum) to 30 (maximum) points, with the highest prevalence of 29 points and 30 points. There was a significant correlation between the BRAZTEL-MMSE and the MMSE (p<0.001), where high scores on the BRAZTEL-MMSE were associated with similarly high scores on the MMSE (rho=0.354; p-value<0.001).

**Table 2:** Frequency distributions of BRAZTEL-MMSE and MMSE scores.

Variables		n=190	%	Mean	SD	Min.	Median	Max.
BRAZTEL		190	100.00	20.47	1.39	15.00	21.00	22.00
	15	1	0.53					
	16	2	1.05					
	17	3	1.58					
	18	12	6.32					
	19	24	12.63					
	20	37	19.47					
	21	63	33.16					
	22	48	25.26					
MMSE		190	100.00	28.52	1.56	23.00	29.00	30.00
	23	1	0.53					
	24	1	0.53					
	25	8	4.21					
	26	16	8.42					
	27	19	10.00					
	28	30	15.79					
	29	48	25.26					
	30	67	35.26					

Abbreviations: SD, standard deviation; Min., minimum; Max., maximum; Braztel-MMSE, Brazilian telephone version of the Mini-Mental State Examination.

**Figure 2:**
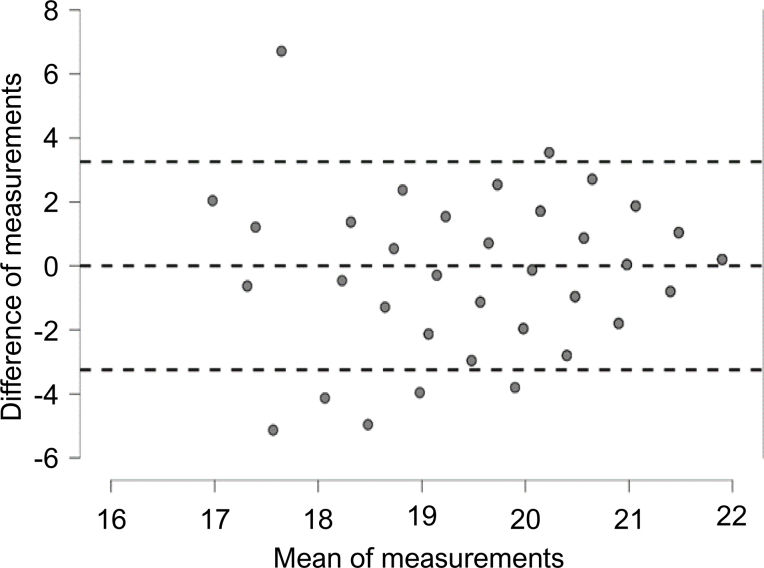
Bland-Altman plot analysis.

Lastly, analysis of the Bland-Altman plot (Figure 2) revealed satisfactory concordance between the two cognitive assessment methods, with a mean difference of 0.002 between scores [+1.96 standard deviation (SD)=3.25; −1.96 SD=-3.25].

## DISCUSSION

The present study compared the cognitive performance of older adults, as assessed by the BRAZTEL-MMSE applied by telephone and by the traditional MMSE applied in person. The results showed a positive correlation between the two scales, indicating that both provide a reliable measure of the cognitive performance of older adults.

The study method adds to the robustness and reliability of the investigation, where the initial sample of 578 participants allowed a broad analysis, while the subsample of 190 participants provided a more in-depth comparison of the BRAZTEL-MMSE and traditional MMSE scales. Moreover, the inclusion of highly educated, socially engaged older adults introduced an extra dimension to the study, since these factors may be associated with greater cognitive reserve, hampering the detection of mild decline^8^. This specificity of the sample helped provide a better understanding of the applicability of the tool in distinct population groups and of the need to refine the cut-offs for different educational and social profiles^9^.

These findings represent an important contribution to the literature, amid a dearth of studies comparing the accuracy of cognitive assessments applied by phone and in person^11,12^. Such studies are important to validate the application of scales via telephone, because most investigations center on administration via videoconferencing as opposed to telephone^13^.

In this context, Liu et al.^14^ described patient factors associated with the use of different modalities of virtual care. Analyzing data obtained from a sample of 330 participants (mean age 83 years), the authors found several limitations to using the virtual approach. Results showed that frail participants and those without a caregiver to assist during assessment had less access to videoconference-based consultations.

In this regard, telephone-based assessments might offer broader screening. Tools such as the BRAZTEL-MMSE provide access to a greater number of people in a shorter timeframe, overcoming geographical barriers and enabling trained professionals to carry out cognitive assessments of individuals who would otherwise not have access to these services. This approach is especially useful in regions with poor infrastructure or in situations where the individuals being assessed have impaired mobility. In addition, the use of such instruments can help optimize the use of available resources, enabling more effective screening without reducing the reliability of results^15^.

The literature reports a significant increase in remote assessment, particularly since the COVID-19 pandemic. The outbreak drove the development and validation of scales adapted for remote use, meeting the need for continuity of health care, despite restricted mobility and social distancing. This trend expanded access to cognitive assessments and highlighted the importance of remote tools that offer the same accuracy and reliability as their in-person counterparts, advancing the democratization of health care^12,16^. It is important to note, however, that the present study sample comprised cognitively healthy older adults, limiting the generalization of results.

With the same objective, Watt et al.^12^ conducted a systematic review to compare the diagnostic accuracy of virtual to in-person cognitive assessments and tests, and to identify barriers to implementing virtual cognitive assessment. The researchers found 121 studies describing the reliability and accuracy of virtual and in-person cognitive assessment tests. The authors concluded that, although virtual tests prove effective for screening cognitive decline and dementia, there are gaps in the diagnostic certainty of virtual assessments.

The findings of the study by Desmond et al.^17^ showed that the Telephone Interview for Cognitive Status (TICS) is a reliable tool for remote screening. Both the TICS and the Braztel-MMSE appeared to provide consistent results comparable to traditional standardized tests such as the MMSE. These approaches underscore the role of telephone screening tools in improving access and accuracy of cognitive assessments, especially for individuals with difficulty accessing services in person. Thus, these tools extend the scope of care delivery and have broad application in clinical and research settings.

Notably, the validity of remote cognitive tools, such as the BRAZTEL-MMSE, shows potential for broadening access to dementia screening, particularly in populations facing geographic or mobility limitations. The results of Lipton et al.^18^ showed that a specific telephone screening tool provided high sensitivity and specificity, making it a valid alternative to conventional methods. Nevertheless, it is important to recognize that, despite its effectiveness, remote screening cannot capture all the complexities of the diagnosis, particularly in more advanced cases. Therefore, while increased use of tools such as the Braztel-MMSE is promising, their administration should be balanced carefully with in-person assessment to ensure accurate and complete diagnoses.

In terms of the state of the art, this study emerges as part of a growing interest in technological approaches for cognitive assessments, broadening access to these assessments. Moreover, the study reveals the need for further investigations exploring the accuracy of virtual remote tests involving different conditions and populations. The study makes valuable contributions in validating a telephone-based screening tool and highlighting the importance of public policies. These policies can promote digital inclusion and equitable access to health care for all age groups and sociodemographic levels.

A limitation found when administering cognitive assessment tools by telephone is the difficulty in controlling the environment, where a number of variables can impact the quality of the assessment, such as distractions, unpermitted notes, and outside assistance^7^. The present study has some limitations, such as the homogeneity of the sample in terms of years of education, with the inclusion of predominantly highly educated individuals. In this respect, outcomes would have been better reported if the study had included, for example, a comparison between high- and low-educated groups of older adults.

Another issue involves the 18-month interval between application of the telephone assessment and the in-person assessment. Application of the scales with a shorter time interval may have more accurately captured the performance of participants, as per Camozzato et al.^5^, who administered the scales with an interval of 48–72 h. Future studies should include more diverse samples of people with different educational levels and sociodemographic profiles. In addition, the time gap between applying the in-person and telephone-based assessments should be made shorter, while longitudinal studies can also be conducted with multiple applications of the scales over time.

Despite the limitations outlined, these results confirm that the BRAZTEL-MMSE relative to the MMSE is effective for assessing the cognitive performance of highly educated socially engaged older individuals. This performance suggests the potential of the BRAZTEL-MMSE scale for use in programs such as health monitoring and clinical trials.

## Data Availability

The datasets generated and/or analyzed during the current study are available from the corresponding author upon reasonable request.
