# Supplementary material for: Mini-Mental State Examination for telephone use in highly educated and socially active older adults: a descriptive study
Source: Dement Neuropsychol. 2026 Jan 23;20:e20250365. doi: 10.1590/1980-5764-DN-2025-0365 (PMC12843192; doi:10.1590/1980-5764-DN-2025-0365)
Supplement: Supplementary Material 1 [file 1980-5764-dn-20-e20250365-Suppl01.docx]

**Supplementary Material 1**

**Sugestões para o uso do mini-exame do estado mental no Brasil conforme o modelo de Brucki et al. (2003).**

| Orientação temporal (5 pontos) | 1. Que dia é hoje? |
| --- | --- |
|  | 1. Em que mês estamos? |
|  | 1. Em que ano estamos? |
|  | 1. Em que dia da semana estamos? |
|  | 1. Qual a hora aproximada? (considere correta a variação de mais ou menos uma hora) |
| Orientação espacial (5 pontos) | 1. Em que local nós estamos? (consultório, dormitório, sala ñ apontando para o chão) |
|  | 1. Que local é este aqui? (apontando ao redor num sentido mais amplo: hospital, casa de repouso, própria casa). |
|  | 1. Em que bairro nós estamos ou qual o nome de uma rua próxima. |
|  | 1. Em que cidade nós estamos? |
|  | 1. Em que Estado nós estamos? |
| Memória imediata (3 pontos): | Eu vou dizer três palavras e você irá repeti-las a seguir: carro, vaso, tijolo (dê 1 ponto para cada palavra repetida acertadamente na 1ª vez, embora possa repeti-las até trÍs vezes para o aprendizado, se houver erros). Use palavras não relacionadas |
| Cálculo (5 pontos): | Subtração de setes seriadamente (100-7, 93-7, 86-7, 79-7, 72-7, 65). Considere 1 ponto para cada resultado correto. Se houver erro, corrija-o e prossiga. Considere correto se o examinado espontaneamente se autocorrigir. |
| Evocação das palavras (3 pontos): | Pergunte quais as palavras que o sujeito acabara de repetir. |
| Nomeação (2 pontos): | peça para o sujeito nomear os objetos mostrados (relógio, caneta). |
| Repetição (1 ponto): | Vou lhe dizer uma frase e quero que você repita depois de mim: “Nem aqui, nem ali, nem lá”. Considere somente se a repetição for perfeita |
| Comando (3 pontos): | Pegue este papel com a mão direita (1 ponto), dobre-o ao meio (1 ponto) e coloque-o no chão (1 ponto). Se o sujeito pedir ajuda no meio da tarefa não dê dicas. |
| Leitura (1 ponto): | Mostre a frase escrita “FECHE OS OLHOS” e peça para o indivíduo fazer o que está sendo mandado. Não auxilie se pedir ajuda ou se só ler a frase sem realizar o comando. |
| Frase (1 ponto): | Peça ao indivíduo para escrever uma frase. Se não compreender o significado, ajude com: alguma frase que tenha começo, meio e fim; alguma coisa que aconteceu hoje; alguma coisa que queira dizer. Para a correção não são considerados erros gramaticais ou ortográficos. |
| Cópia do desenho (1 ponto): | Mostre o modelo e peça para fazer o melhor possível. Considere apenas se houver 2 pentágonos interseccionados (10 ‚ângulos) formando uma figura de quatro lados ou com dois ângulos  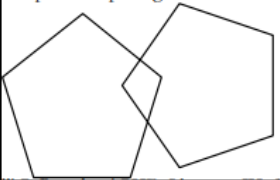 |
