# Supplementary material for: Mini-Mental State Examination for telephone use in highly educated and socially active older adults: a descriptive study
Source: Dement Neuropsychol. 2026 Jan 23;20:e20250365. doi: 10.1590/1980-5764-DN-2025-0365 (PMC12843192; doi:10.1590/1980-5764-DN-2025-0365)
Supplement: Supplementary Material 2 [file 1980-5764-dn-20-e20250365-Suppl02.docx]

**Supplementary Material 2**

**Mini-Exame do Estado Mental Telefônico Brasileiro (Camozzato et al., 2011)**

| **Pergunta** | **Certo** | **Errado** |
| --- | --- | --- |
| **Orientação Temporal** | | |
| 1. Que dia é hoje? | 1 | 0 |
| 1. Em que mês estamos? | 1 | 0 |
| 1. Em que ano estamos? | 1 | 0 |
| 1. Em que dia da semana estamos? | 1 | 0 |
| 1. Qual a hora aproximada? (considere correta a variação de mais ou menos uma hora) | 1 | 0 |
| **Orientação Espacial** | | |
| 1. Em que local o(a) Sr.(a) está?   (considere correta se a resposta se referir ao aposento da residência ou se for "em casa" ou "em meu apartamento") | 1 | 0 |
| 1. Em que bairro/rua o(a) Sr.(a) está?   (obter a informação previamente) | 1 | 0 |
| 1. Em que cidade o(a) Sr.(a) está? | 1 | 0 |
| 1. Em que estado o(a) Sr.(a) está? | 1 | 0 |
| **Registro de memória imediata** | | |
| 1. Vou dizer 3 palavras, e você irá repeti-las a seguir: **CARRO, VASO, TIJOLO**. (caso não consiga, repita no máximo 3 vezes para aprendizado. Pontue a primeira tentativa). | | |
| Carro | 1 | 0 |
| Vaso | 1 | 0 |
| Tijolo | 1 | 0 |
| **Atenção e Cálculo** | | |
| 1. Gostaria que você me dissesse quanto é: 100 - 7; 93 - 7; 86 - 7, 79 - 7; 72 - 7 (65) (se houver erro corrija-o e prossiga. Considere correto se o examinado espontaneamente se corrigir). | | |
| 100 - 7 | 1 | 0 |
| 93 - 7 | 1 | 0 |
| 86 - 7 | 1 | 0 |
| 79 - 7 | 1 | 0 |
| 72 - 7 (65) | 1 | 0 |
| **Memória e evocação** | | |
| 1. Você consegue se lembrar das 3 palavras que lhe pedi que repetisse agora a pouco? | | |
| Carro | 1 | 0 |
| Vaso | 1 | 0 |
| Tijolo | 1 | 0 |
| **Linguagem** | | |
| 1. Qual é o nome do objeto através do qual estamos falando? / Qual é o nome do objeto que estamos usando para falarmos um com o outro? | 1 | 0 |
| 1. Preste atenção: vou lhe dizer uma frase e quero que repita depois de mim NEM AQUI, NEM ALI, NEM LÁ. (considere somente se a repetição for perfeita). | 1 | 0 |

**Pontuação final: ____/22**
